# Antibody Persistence After Primary SARS-CoV-2 Infection and Protection Against Future Variants Including Omicron in Adolescents: National, Prospective Cohort Study

**DOI:** 10.1097/INF.0000000000003890

**Published:** 2023-03-01

**Authors:** Felicity Aiano, Georgina Ireland, Frances Baawuah, Joanne Beckmann, Ifeanyichukwu O. Okike, Shazaad Ahmad, Joanna Garstang, Andrew J. Brent, Bernadette Brent, Ray Borrow, Ezra Linley, Sammy Ho, Christine Carr, Maria Zambon, John Poh, Lenesha Warrener, Gayatri Amirthalingam, Kevin E. Brown, Mary E. Ramsay, Katja Hoschler, Shamez N. Ladhani

**Affiliations:** From the * Immunisation and Vaccine Preventable Diseases Division, UK Health Security Agency; † East London NHS Foundation Trust, London; ‡ University Hospitals of Derby and Burton NHS Foundation Trust, Derby; § Manchester University NHS Foundation Trust, Manchester; ¶ Birmingham Community Healthcare NHS Trust, Aston; ‖ Nuffield Department of Medicine, Oxford University Hospitals NHS Foundation Trust; ** University of Oxford, Oxford; †† UK Health Security Agency, Manchester Royal Infirmary, Manchester; ‡‡ Paediatric Infectious Diseases Research Group, St. George’s University of London, London, United Kingdom.

**Keywords:** school, antibody, SARS-CoV-2

## Abstract

Antibodies are a measure of immunity after primary infection, which may help protect against further SARS-CoV-2 infections. They may also provide some cross-protection against SARS-CoV-2 variants. There are limited data on antibody persistence and, especially, cross-reactivity against different SARS-CoV-2 variants after primary infection in children.

**Methods:**

We initiated enhanced surveillance in 18 secondary schools to monitor SARS-CoV-2 infection and transmission in September 2020. Students and Staff provided longitudinal blood samples to test for variant-specific SARS-CoV-2 antibodies using in-house receptor binding domain assays. We recruited 1189 students and 1020 staff; 160 (97 students, 63 staff) were SARS-CoV-2 nucleocapsid-antibody positive at baseline and had sufficient serum for further analysis.

**Results:**

Most participants developed sustained antibodies against their infecting [wild-type (WT)] strain as well as cross-reactive antibodies against the Alpha, Beta and Delta variants but at lower titers than WT. Staff had significantly lower antibodies titers against WT as cross-reactive antibodies against the Alpha, Beta and Delta variants than students (all *P* < 0.01). In participants with sufficient sera, only 2.3% (1/43) students and 17.2% (5/29) staff had cross-reactive antibodies against the Omicron variant; they also had higher antibody titers against WT (3042.5; 95% confidence interval: 769.0–12,036.2) than those who did not have cross-reactive antibodies against the Omicron variant (680.7; 534.2–867.4).

**Conclusions:**

We found very high rates of antibody persistence after primary infection with WT in students and staff. Infection with WT induced cross-reactive antibodies against Alpha, Beta and Delta variants, but not Omicron. Primary infection with WT may not be cross-protective against the Omicron variant.

Children are more likely to remain asymptomatic after SARS-CoV-2 infection or develop a mild, self-limiting illness than adults.^1^ In the United Kingdom, the first COVID-19 cases were identified in late January 220, with endemic cases rising rapidly in March 2020, leading to the first national lockdown, including school closures.^2^ Case numbers peaked in April and then declined, allowing some school years to partially reopen for in-person teaching in June 2020 and fully reopen for all school years in September 2020, *albeit* with extensive infection control measures.^3^

To investigate SARS-CoV-2 infection and transmission in educational settings, the UK Health Security Agency (UKHSA, formerly Public Health England) initiated sKIDs serosurveillance in primary and secondary schools across England.^4–6^ Participation involved sequential blood sampling for SARS-CoV-2 antibodies and other immune function tests. SARS-CoV-2 antibodies provide a robust measure of prior exposure to the virus and capture both symptomatic and asymptomatic infection. In vaccinated individuals, antibody testing can distinguish between natural immunity which induces SARS-CoV-2 nucleocapsid (N-antibody) and Spike protein (S-antibody) antibodies, compared with vaccine-induced immunity which only induces S-antibodies.

Antibodies are also a measure of immunity after primary infection, which may help protect against SARS-CoV-2 reinfections. They may also provide some cross-protection against SARS-CoV-2 variants with similar surface antigen epitopes, especially those within the spike protein and, more specifically, within the angiotensin-converting enzyme 2 receptor binding domain (RBD) of the spike protein, which is a major SARS-CoV-2 virulence factor.^7^

In England, the initial SARS-CoV-2 wild-type (WT) strain responsible for the first wave of the COVID-19 pandemic was rapidly replaced by the more transmissible Alpha (B.1.1.7) variant since December 2020, the Delta (B.1.617.2) variant since April 2021 and the Omicron (B.1.1.529) variant since December 2021. In addition to antigenic drift between the different variants,^8^ waning immunity resulting in lower antibody titers after primary infection may also increase the risk of reinfection, which may vary with the different variants. With seasonal coronaviruses, for example, children are known to have multiple and recurrent infections, which may at least in part be explained by antigenic drift and antibody waning.^9,10^

Unlike adults, there are limited data on antibody persistence and, especially, cross-reactivity against SARS-CoV-2 variants after primary infection in children.^11^ Through the sKIDsPLUS serosurveillance in secondary schools, we identified students and staff who had been infected with WT during the first wave of the pandemic and had measurable SARS-CoV-2 antibodies when they returned to school in September 2020.^12^ Here, we assessed antibody persistence for up to 12 months after their initial infection as well as trends in antibody cross-reactivity against the Alpha, Beta, Delta and Omicron variants using validated, in-house variant-specific RBD assays. Antibodies against SARS-CoV-2 RBD correlate most closely with virus neutralizing activity,^13,14^and clinical protection,^15^ compared to other regions of the SARS-CoV-2 spike protein, most likely because RBD antibodies directly interfere with binding to the host angiotensin-converting enzyme 2 receptor and, therefore, preventing viral entry into the host cell.

## METHODS

The COVID-19 Surveillance in Secondary School KIDs (sKIDsPLUS) protocol is available online (https://www.gov.uk/guidance/covid-19-paediatric-surveillance),^16^ and SARS-CoV-2 antibody seroprevalence results have been published.^5,12,17^ Briefly, secondary schools in West London, East London, Hertfordshire, Derbyshire, Greater Manchester and Birmingham, where our pediatric investigation teams were assembled, were approached to participate in sKIDsPLUS. Headteachers in participating schools emailed the study information pack to staff, parents of students <16 years and directly to students ≥16 years of age. Participants ≥16 years or the parent/guardian of ≤16 years old provided informed consent online via SnapSurvey, completed a short questionnaire on COVID-19 symptoms and confirmed infection before the sampling day or shortly afterward. A team of clinicians, nurses, phlebotomists and administrative staff attended the school on sampling days, and a nasal swab and blood sample were taken from each participant. Samples were taken at the start (round 1: September 22–October 17, 2020) and end (round 2: December 3–17, 2020) of the autumn term of the 2020/2021 academic year, and when the schools reopened in March 2021 (round 3: March 23–April 21, 2021).

### Antibody Testing

Serology was initially performed on the Abbott Architect, using a chemiluminescent microparticle immunoglobulin G (IgG) immunoassay targeting the nucleoprotein (N) (SARS-CoV-2 IgG, Abbott Commerce Chicago, Illinois) with a seropositivity cutoff value of 0.8 (henceforth referred to as Abbott N assay).^18^ Sera from seropositive participants in round 1 who also participated in rounds 2 and 3 were subsequently tested for RBD antibodies using an in-house indirect IgG RBD assay for the infecting WT strain.^19^ Commercial RBD subunit was purchased from SinoBiological Inc. (Beijing, P.R. China) and expressed in HEK293 cell culture with a C-terminal mouse Fc tag (Arg319-Phe541(V367F);#YP_009724390.1). Nunc MaxiSorp flat-bottomed, polystyrene 96-well microtiter plates were coated by diluting 20 ng recombinant protein/well in sterile phosphate-buffered saline; pH7.2 ± 0.05 (-CaCl2, -MgCl2), (GIBCO, Thermo Fischer, Waltham, Massachusetts) at 4–8°C for a minimum of 16 hours. After washing and blocking coated plates, sera were diluted at a final dilution factor of 1/100. IgG binding on the plate surface was detected with an anti-Human IgG−horseradish peroxidase antibody conjugate (Sigma Aldrich, St Louis) and detected with 3,3′, 5,5′-Tetramethylbenzidine (Europa Bioproducts Ltd, Ipswich, United Kingdom). Samples were analyzed in duplicate and optical density (OD450) data were evaluated by dividing average OD450 values for individual samples by the average OD450 of a known calibrator with negative antibody levels (T/N ratio). The seropositivity cutoff index threshold was 5. The samples were analyzed together to minimize assay-to-assay variation, with controls included to monitor assay performance and consistency.

For variant serology, the standard method was modified as follows: endpoint titers were determined on Nunc MaxiSorp flat-bottomed, polystyrene 96-well microtiter plates which were coated 20ng variant protein/well in sterile phosphate-buffered saline; pH7.2 ± 0.05 (-CaCl_2_, -MgCl_2_).^19^ The constructs for the Alpha, Beta, Delta and Omicron variants were obtained from the same commercial supplier as the WT strain (SinoBiological Inc.), all reagent and dilutions used in the original method remained, but the analysis was performed by serially diluting each serum sample starting at 1:100 (6-fold with the highest dilution achieved 129600) to determine antibody titers. Samples were analyzed with all antigens (including WT for comparison) in the presence of known positive controls (individuals with confirmed SARS-CoV-2 infection) and a calibrator sample (“negative” added to four wells; collected prior to the pandemic). Titers are expressed as serum fold-dilution required to achieve a T/N (test OD to negative OD) of 5 (T/N = 5 serves as cutoff for positive samples) by xy interpolation from the RBD data series (dilution, x vs. OD450, y). Samples below this cutoff in the initial dilution were expressed as <100 and recoded to 75 for analysis. The in-house variant RBD assays have demonstrated high correlation with in vitro virus neutralizing activity, particularly for the Alpha (Spearman’s rho = 0.80) and Delta (r = 0.86) variants but lower for the Beta variant (r = 0.49).^20^

## STATISTICAL ANALYSIS

Data were analyzed using Stata SE (version 15.1). RBD variant antibody geometric mean titers (GMTs) were calculated with 95% confidence intervals (CIs). GMTs between variants, between staff and students, and between sampling rounds were statistically different when 95% CIs did not overlap. Geometric mean ratios (GMRs) of responses were estimated using mixed regression models on log responses to compare (1) RBD variant responses of staff and students for each variant at each time point; (2) RBD variant responses between the three testing rounds for staff and students separately; and (3) GMTs of staff and students with and without antibody boosting at the final sampling. Antibody boosting between rounds 2 and 3 was explored by assessing how many students had a 100% increase in Alpha variant RBD titers (the dominant circulating variant at the time of sampling). chi-square and Fisher exact tests were used to compare categorical variables.

## RESULTS

**FIGURE 1.**
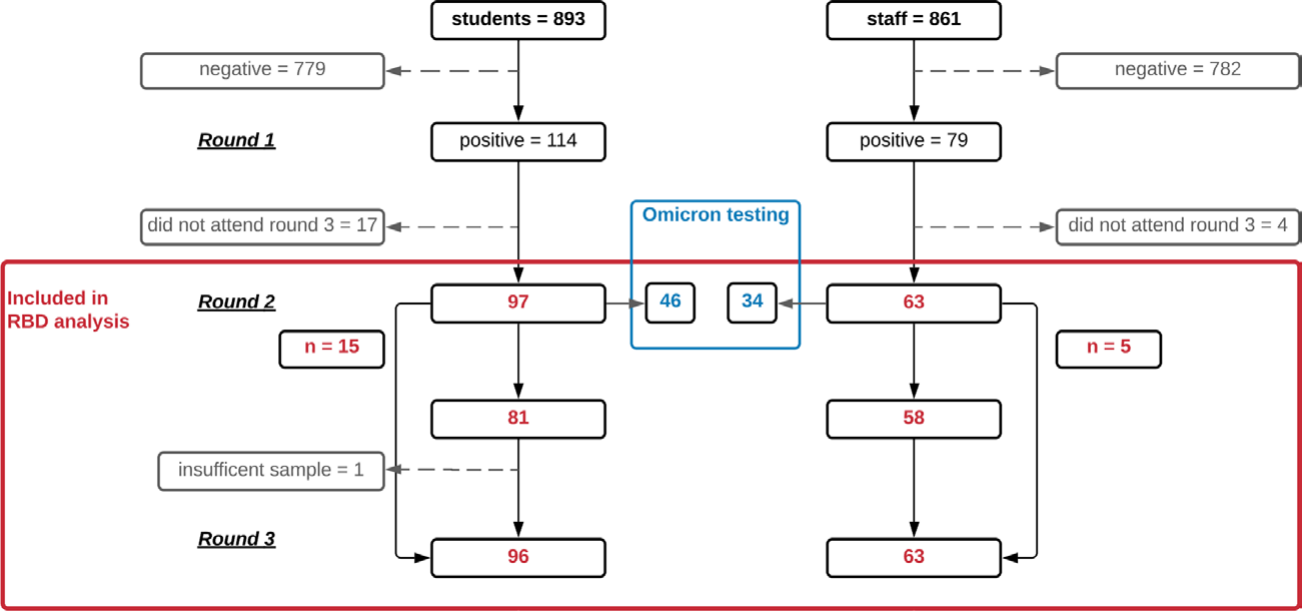
Identification and selection of study sample participants from sKIDs study population.

In September 2020, 2209 participants (1189 students and 1020 staff) were recruited to sKIDsPLUS and 193 (8.7%) were SARS-CoV-2 N-antibody positive. Of these, 160 (82.9%) also attended rounds 2 and 3 (n = 140) or missed round 2 but attended round 3 (n = 20) (Table 1; Fig. 1). Therefore, samples from 97 students (median age, 14 years; range 11–17 years) and 63 staff (median age, 47 years; range, 23–64 years) were included in the final analysis, comprising 71 (44.4%) males and 89 (55.6%) females. When compared with staff, a higher proportion of students were RBD antibody seropositive against WT, Alpha, Beta and Delta variants at all time points (Table 2).

**TABLE 1.** Demographic Characteristics of sKIDs PLUS Participants, Students and Staff, From 20 Secondary Schools in England Who Tested Positive for N Antibodies on the Abbott N Assay in September/October 2020 and Included in Analysis

	Total		Students		Staff	
	n	Percent, %	n	Percent, %	n	Percent, %
Sex						
Male	71	44.4	44	45.4	27	42.9
Female	89	55.6	53	54.6	36	57.1
Age category, yr						
11–12	28	17.5	28	28.9		
13–14	46	28.8	46	28.8		
15–17	23	14.4	23	14.4		
20–29	8	5.0			8	12.7
30–39	13	8.1			13	20.6
40–49	17	10.6			17	27.0
50 +	25	15.6			25	39.7
Ethnicity						
White	100	62.5	55	56.7	45	7.1
Black	14	8.8	7	7.2	7	1.1
Asian	29	18.1	21	21.6	8	1.3
Mixed	8	5.0	5	5.2	3	0.5
Other	9	5.6	9	9.3	0	0.0
PCR diagnosed	11	6.9	8	8.2	3	0.5
Total	160		97		63	

**TABLE 2.** Positivity and Geometric Mean of WT, Alpha, Delta, Beta Variant RBD Projected Titers by Round and Participant Type, and GMR of Staff and Student RBD Projected Titers for Each Variant and Round of Testing

		Geometric Mean						GMR of Staff vs Student*		
		Students			Staff					
		n/N (%)	GM†	95% CI	n/N (%)	GM†	95% CI	GMR	95% CI	*P* ‡
Round 1	WT	92/97 (94.8)	844.3	702.9–1014.2	51/63 (81.0)	522.3	373.9–729.8	0.62	0.44–0.87	0.007
	Alpha	92/97 (94.8)	761.4	634.4–913.8	51/62 (82.3)	483.1	352.3–662.5	0.63	0.45–0.89	0.008
	Delta	92/97 (94.8)	634.0	532.8–754.4	46/62 (74.2)	375.4	272.1–518.1	0.59	0.43–0.82	0.002
	Beta	86/97 (88.7)	419.3	350.9–501.1	37/62 (59.7)	243.8	178.4–333.3	0.58	0.42–0.81	0.001
Round 2	WT	77/81 (95.1)	619.7	511.4–750.9	48/58 (82.8)	449.8	326.6–619.6	0.73	0.51–1.03	0.070
	Alpha	77/81 (95.1)	654.0	536.1–797.9	47/58 (81.0)	444.9	323.9–611.0	0.68	0.48–0.97	0.031
	Delta	76/81 (93.8)	507.4	420.4–612.6	43/58 (74.1)	323.3	237.8 - 439.7	0.64	0.46–0.89	0.009
	Beta	71/81 (87.7)	324.0	271.7–386.4	33/58 (56.9)	208.7	156.1–279.0	0.64	0.47–0.89	0.006
Round 3	WT	93/96 (96.9)	872.8	725.9–1049.4	59/63 (93.7)	3116.0	1802.4–5386.8	3.57	2.19–5.82	<0.001
	Alpha	93/96 (96.9)	943.0	778.1–1142.9	59/63 (93.7)	3172.8	1831.3–5497.1	3.36	2.05–5.52	<0.001
	Delta	93/96 (96.9)	748.2	622.1–899.9	58/63 (92.1)	2451.1	1418.3–4236.1	3.28	2.01–5.34	<0.001
	Beta	89/96 (92.7)	484.7	404.5–580.7	53/63 (84.1)	1464.0	838.6–2556.0	3.02	1.84–4.95	<0.001

* Student is ref category.

† Negative projected titer which is recoded as 75.

‡ *P* value calculated using chi^2^ and Fisher exact tests.

### Round 1

**FIGURE 2.**
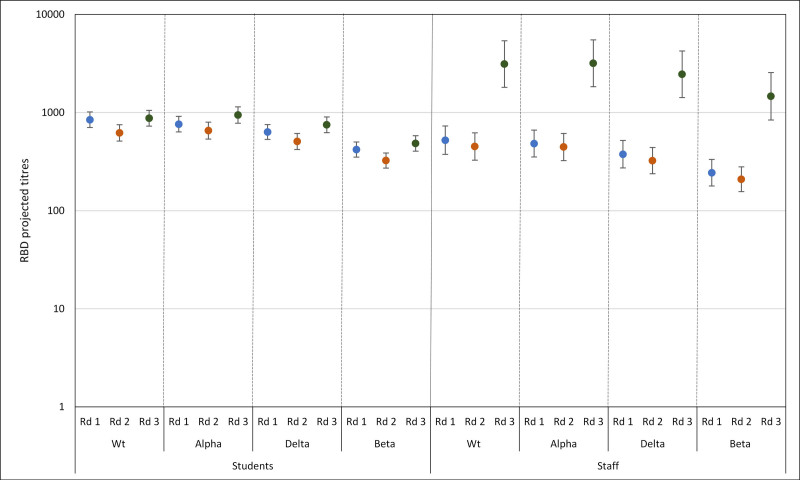
Geometric mean, with 95% CIs, RBD antibody titers for staff and students by variants and rounds (blue = round 1, orange = round 2, green = round 3).

Among N-antibody seropositive students, SARS-CoV-2 variant RBD antibody GMTs were lower for Alpha, Beta and Delta compared with WT, but this was only statistically significant for Beta [844.3 (95% CI, 702.9–1014.2) vs. 419.3 (95% CI, 350.9–501.1)] (Table 2; Fig. 2). The same pattern was observed for staff, with significantly lower antibody GMTs against Beta [522.3 (95% CI, 373.9–729.8) vs. 243.8 (95% CI, 178.4–333.3)]. When compared with staff, students had significantly higher SARS-CoV-2 RBD antibody titers for WT as well as Alpha, Delta and Beta (GMR of 0.62, 0.63, 0.59 and 0.58, respectively, all *P* < 0.01) (Table 2). For both students and staff, the SARS-CoV-2 variant RBD antibody GMTs against Beta were also significantly lower than against WT, Alpha and, for students, also against Delta.

### Round 2

By December 2020 (round 2), there were 81 students and 58 staff who were seropositive in round 1 and has sufficient serum for testing in round 2. In both students and staff, a similar proportion of participants were seropositive on the variant RBD assays as in round 1. When assessing variant-specific antibody titers, GMTs were lower for WT and the 3 variants in round 2 compared with round 1, but GMRs comparing rounds 1 and 2 for each variant were only significantly lower for WT strain and Beta in students (Table 3). When compared with staff, students had higher RBD antibody GMTs, significantly so Alpha, Beta and Delta (GMR of 0.68, 0.64, 0.64, respectively, all *P* < 0.05) (Table 2; Fig. 2).

**TABLE 3.** GMR of RBD Variant Projected Titers Between Rounds

		Students				Staff			
		GM	GMR	95% CI	*P* *	GM	GMR	95% CI	*P* *
WT	Round 1	844.3	Ref			522.3	Ref		
	Round 2	619.7	0.73	0.56–0.96	0.022	449.8	0.86	0.48–1.55	0.616
	Round 3	872.8	1.03	0.80–1.33	0.798	3116.0	5.97	3.36–10.58	<0.001
Alpha	Round 1	761.4	Ref			483.1	Ref		
	Round 2	654.0	0.86	0.66–1.13	0.272	444.9	0.92	0.52–1.64	0.781
	Round 3	943.0	1.24	0.96–1.61	0.106	3172.8	6.57	3.72–11.60	<0.001
Delta	Round 1	634.0	Ref			375.4	Ref		
	Round 2	507.4	0.80	0.62–1.04	0.091	323.3	0.86	0.48–1.53	0.612
	Round 3	748.2	1.18	0.92–1.51	0.189	2451.1	6.53	3.71–11.50	<0.001
Beta	Round 1	419.3	Ref			243.8	Ref		
	Round 2	324.0	0.77	0.60–1.00	0.047	208.7	0.86	0.48–1.52	0.597
	Round 3	484.7	1.16	0.91–1.47	0.243	1464.0	6.00	3.42–10.55	<0.001

* *P* value calculated using chi-square and Fisher exact tests.

### Round 3

In April 2021 (round 3), a similar and very high proportion of students (96.9% for WT, Alpha and Delta; 92.9% for Beta) were seropositive on the variant RBD assays as in rounds 1 and 2 (Table 2). Following a reduction in WT and variant-specific GMTs for students in round 2, GMTs in round 3 for all 4 strains were similar to those observed in round 1, with small (GMRs 1.41 to 1.50) but significant (*P* < 0.011 for all comparisons) increases in antibody titers for WT and all three variants between rounds 2 and 3 (Table 3 and Table 1, Supplemental Digital Content 1, http://links.lww.com/INF/E968; Fig. 2).

Among staff, a higher proportion were seropositive for WT and all 3 variants in round 3 compared with round 2, significantly so for Delta (c^2^, *P* = 0.013) and Beta (c^2^, *P* = 0.001). Additionally, compared with students, staff had much higher antibody GMTs against WT and the 3 variants because most staff had been vaccinated against COVID-19 by round 3. Among staff, GMTs were significantly (*P* < 0.001 for all) higher (GMRs 6.93–7.58) in round 3 than in round 2 for WT and all 3 variants (Table 3 and Table 1, Supplemental Digital Content 1, http://links.lww.com/INF/E968). In round 3, overall SARS-CoV-2 RBD antibody GMTs in both staff and students were highest for Alpha followed by WT, Delta and Beta (Table 2).

Between rounds 2 and 3, Alpha was the main circulating variant in England. Consequently, 15% (12/80) of students and 51.7% (30/58) of staff had a >100% increase in antibody titers against Alpha between rounds 2 and 3, including 1 student and 7 staff who had no detectable antibodies against the Alpha in round 2.

### Omicron Variant

A subset of seropositive samples from round 1 with sufficient sera (46 students, 34 staff) were tested on the Omicron RBD assay. Of these, 3 students and 5 staff who tested negative in the RBD assay against WT also tested negative on the Omicron RBD assay. In those who tested positive in the WT RBD assay, only 2.3% (1/43) of students and 17.2% (5/29) staff also tested positive on the Omicron RBD assay. Among the 6 Omicron RBD-positive participants, the SARS-CoV-2 RBD antibody GMTs against WT were 3042.5 (95% CI, 769.1–12,036.2) compared with 680.7 (95% CI, 534.2–867.4) in those who tested negative on the Omicron RBD assay.

## DISCUSSION

In September 2020, we found that secondary school students were more likely to be seropositive and have higher antibody titers than staff against their initial infecting WT strain. Students also had higher cross-reactive antibodies than staff against the Alpha, Beta and Delta variants, which had yet to emerge at the time of blood sampling. Although cross-reactive RBD antibody GMTs were lower for the variants compared with WT, this was only statistically significant for the Beta variant in both students and staff. Antibody titers against WT and cross-reactive antibodies against Alpha, Beta and Delta variants in students and staff declined from round 1 (September 2020) to round 2 (December 2020) but increased by round 3 (April 2021). In students who were not eligible for COVID-19 vaccination until September 2021, this was most likely because of natural boosting through exposure to the Alpha variant which emerged in December 2020. This is reflected in the greatest increase in antibody GMTs against the Alpha variant compared with WT or the other 2 variants between rounds 2 and 3. In staff, additionally, COVID-19 vaccination became available in January 2021, and was associated with a >4-fold rise in antibody GMTs not only against WT (on which the vaccines were based) but also against the Alpha, Beta and Delta variants. Finally, when the initial sera from seropositive participants in September 2020 were tested against the Omicron variant in a variant-specific RBD assay, only 8% (6/75) tested positive. Those with cross-reactive antibodies against the Omicron variant had much higher antibody GMTs against their infecting WT strain compared with those who without cross-reactive antibodies against the Omicron variant.

Compared with binding antibody titers, a number of studies have reported a stronger correlation between protection against COVID-19 and neutralizing SARS-CoV-2 antibodies.^19,21,22^ At UKHSA, we developed an in-house RBD assay against WT,^22^ and the major SARS-CoV-2 variants including Omicron, and showed that they correlate with variant-specific in vitro virus neutralizing activity,^20^ which in turn correlates with clinical protection.^23^ RBD assays have several advantages over virus neutralization assays in that they are quicker to perform, require less serum and less personnel time, are rapidly adaptable to new variants and are substantially cheaper, without the added element of safety and containment requirements for live-virus studies.

Using these variant-specific RBD assays, we found that most adolescents and adults who were infected with WT early in the first pandemic wave developed and retained cross-reactive antibodies against the Alpha, Beta and Delta variants for up to 12 months after infection. While antibody levels were generally lower against these variants, this reduction was only significant for the Beta variant, which is the most genotypically, antigenically and phenotypically diverse of the 3 variants.^24^ These findings are consistent with adult studies, including our own studies in adults,^19^ and in younger children.^25^ On the other hand, infection with WT appeared to be less cross-reactive against the Omicron variant, which was first identified in South Africa in November 2021 and associated with high rates of infection and reinfection. Subsequent studies confirmed that, while neutralization of ancestral virus was much higher in infected and vaccinated participants compared to vaccinated-only participants, both groups showed a 22-fold escape from vaccine-elicited neutralization by the Omicron variant.^26^

At UKHSA, we rapidly developed and validated an in-house Omicron RBD assay and showed a strong correlation between Omicron RBD antibodies and in vitro virus neutralization with the same variant.^20,27^ Consistent with early reports from South Africa, we also found that serum from adolescents and adults with primary WT infection were poorly cross-reactive against Omicron. We did not test sera from further rounds because of the very low positivity rates against Omicron in round 1. Notably, the small proportion of participants with cross-reactive antibodies against Omicron also had higher antibody levels against WT compared with those who tested negative, suggesting that higher binding antibody levels may provide better protection against SARS-CoV-2 variants and that waning antibodies may increase the risk of infection with new variants over time. This observation may explain why a third dose of COVID-19 vaccine, which was based on WT, provided higher in vitro virus neutralizing antibody,^20^ and better protection against symptomatic disease with Omicron.^28^ This protection was in addition to the well-described protection against the Alpha and Delta, which were the main variants circulating in the United Kingdom before the emergence of Omicron.^29^

In England, Omicron cases increased across all age groups in December 2021 and, in children, surged in January 2022 when students returned to school with very limited in-school mitigations in place.^30^ Notably, many of the children who are infected with Omicron had been infected with Delta <3 months previously, highlighting the limited protection offered by previous Delta infection against Omicron in children.^31^ In England, around half the 12- to 15-year-olds had received their first dose of COVID-19 mRNA vaccine by the end of 2021, with a second dose planned around 12 weeks after their first dose.^32^ COVID-19 vaccines are highly effective in reducing hospitalizations and deaths due to COVID-19 but provide limited short-term protection against symptomatic COVID-19, especially due to Omicron, in adults^28^ and adolescents.^29^

Longitudinal follow-up of our cohort allowed us to assess trends in antibodies against variants after natural infection in a cohort of children who were largely unvaccinated during the surveillance period. Antibody levels rise rapidly after acute SARS-CoV-2 infection and then decline gradually. Studies with longer follow-up suggest that antibody levels, including neutralizing antibodies, stabilize after 4 to 6 months postinfection, with neutralizing activity being significantly lower for variants compared with the infecting strain.^33^ In our cohort, antibody levels from round 1 (around 6 months after the first pandemic wave when most participants were infected) declined in round 2 (around 3 months after round 1) for WT and the 3 variants, thus following the trend observed for neutralizing antibodies. Notably, overall titers as well as individual titers in a subset of students (15%) increased by round 3, reaching round 1 levels in students. This is most likely because of natural boosting with the Alpha variant, highlighting a role for circulating strains in maintaining population immunity against the virus.

### Strengths and Limitations

The strength of this study is the longitudinal collection of blood samples from students and staff when they returned to full-time in-person education in England. This allowed us to monitor and compare trends in infection and immunity between adolescents and adults who were infected with WT early in the pandemic and the protection offered against emerging variants over time. A limitation of the study is that we did not have laboratory-confirmation of the participants’ initial infection and, therefore, can only speculate on the timing of their infection. Reassuringly, adolescents and adults develop similar robust and long-lasting immune responses irrespective of their symptom status following infection.^5,34^ Another limitation is that we used RBD assays to assess antibody responses against variants as an alternative to virus neutralization assays but we have shown strong correlation between the two and our findings in adolescents are consistent with the reported literature in adults. Finally, we only assessed antibody titers and cross-reactivity after infection and vaccination. Protection against reinfection and, importantly, against serious disease will also depend T-cell responses,^35^ which we did not assess in our cohort.

## CONCLUSIONS

Our findings add to the limited literature on SARS-CoV-2 immunity in children compared to adults. We found very high levels of antibody persistence after primary infection with WT in adolescents and staff. Using validated in-house variant-specific RBD assays, we found that WT infection resulted in cross-reactive antibodies against the Alpha, Beta and Delta variants, *albeit* at lower levels than WT. Very few, however, had antibodies against Omicron, consistent with high rates of reinfection after primary infection and breakthrough infections after vaccination observed with Omicron.

## ACKNOWLEDGMENTS

*The authors thank the schools, headteachers, staff, families and their very brave children who took part in the sKIDs surveillance.*

## Supplementary Material
