# Supplementary material for: Antibody Persistence After Primary SARS-CoV-2 Infection and Protection Against Future Variants Including Omicron in Adolescents: National, Prospective Cohort Study
Source: Pediatr Infect Dis J. 2023 Mar 1;42(6):496–502. doi: 10.1097/INF.0000000000003890 (PMC10187624; doi:10.1097/INF.0000000000003890)
Supplement: Supplementary file 1 [file inf-42-496-s001.docx]

| **Supplementary Digital Content 1:** Geometric Mean Ratio of RBD variant projected titres between rounds, when compared to Round 2 (reference category) | | | | | | | | | | | | | |
| --- | --- | --- | --- | --- | --- | --- | --- | --- | --- | --- | --- | --- | --- |
|  |  |  |  |  |  |  |  |  |  |  |  |  |  |
|  |  |  |  |  |  |  |  |  |  |  |  |  |  |
|  |  |  | Students | | | |  | Staff | | | |  |  |
|  |  |  | GMT | GMR | 95% CI | p-value |  | GMT | GMR | 95% CI | p-value |  |  |
|  | Wt | Round 1 | 844.3 | 1.36 | 1.05-1.76 | 0.022 |  | 522.3 | 1.16 | 0.65-2.08 | 0.616 |  |  |
|  |  | Round 2 | 619.7 | Ref |  |  |  | 449.8 | Ref |  |  |  |  |
|  |  | Round 3 | 872.8 | 1.41 | 1.08-1.84 | 0.011 |  | 3116.0 | 6.93 | 3.86-12.43 | <0.001 |  |  |
|  | Alpha | Round 1 | 761.4 | 1.16 | 0.89-1.53 | 0.272 |  | 483.1 | 1.09 | 0.61-1.94 | 0.781 |  |  |
|  |  | Round 2 | 654.0 | Ref |  |  |  | 444.9 | Ref |  |  |  |  |
|  |  | Round 3 | 943.0 | 1.44 | 1.10-1.89 | 0.008 |  | 3172.8 | 7.13 | 4.00-12.71 | <0.001 |  |  |
|  | Delta | Round 1 | 634.0 | 1.25 | 0.94-1.62 | 0.091 |  | 375.4 | 1.16 | 0.65-2.07 | 0.612 |  |  |
|  |  | Round 2 | 507.4 | Ref |  |  |  | 323.3 | Ref |  |  |  |  |
|  |  | Round 3 | 748.2 | 1.47 | 1.14-1.91 | 0.003 |  | 2451.1 | 7.58 | 4.26-13.47 | <0.001 |  |  |
|  | Beta | Round 1 | 419.3 | 1.29 | 1.00-1.67 | 0.047 |  | 243.8 | 1.17 | 0.66-2.08 | 0.597 |  |  |
|  |  | Round 2 | 324.0 | Ref |  |  |  | 208.7 | Ref |  |  |  |  |
|  |  | Round 3 | 484.7 | 1.50 | 1.16-1.93 | 0.002 |  | 1464.0 | 7.01 | 3.95-12.45 | <0.001 |  |  |
|  |  |  |  |  |  |  |  |  |  |  |  |  |  |
